# Metabolomics Signature of Plasma Renin Activity and Linkage with Blood Pressure Response to Beta Blockers and Thiazide Diuretics in Hypertensive European American Patients

**DOI:** 10.3390/metabo11090645

**Published:** 2021-09-21

**Authors:** Mai Mehanna, Caitrin W. McDonough, Steven M. Smith, Yan Gong, John G. Gums, Arlene B. Chapman, Julie A. Johnson, Lauren McIntyre, Rhonda M. Cooper-DeHoff

**Affiliations:** 1 Department of Pharmacotherapy and Translational Research and Center for Pharmacogenomics and Precision Medicine, College of Pharmacy, University of Florida, Gainesville, FL 32610, USA; mmehanna@ufl.edu (M.M.); cmcdonough@cop.ufl.edu (C.W.M.); ssmith@cop.ufl.edu (S.M.S.); gong@cop.ufl.edu (Y.G.); jgums@ufl.edu (J.G.G.); julie.johnson@ufl.edu (J.A.J.); 2 Department of Medicine, University of Chicago, Chicago, IL 60637, USA; achapman1@medicine.bsd.uchicago.edu; 3 Department of Molecular Genetics and Microbiology, College of Medicine, University of Florida, Gainesville, FL 32610, USA; mcintyre@ufl.edu

**Keywords:** plasma renin activity, metabolomics, hypertension, blood pressure

## Abstract

Plasma renin activity (PRA) is a predictive biomarker of blood pressure (BP) response to antihypertensives in European–American hypertensive patients. We aimed to identify the metabolic signatures of baseline PRA and the linkages with BP response to β-blockers and thiazides. Using data from the Pharmacogenomic Evaluation of Antihypertensive Responses-2 (PEAR-2) trial, multivariable linear regression adjusting for age, sex and baseline systolic-BP (SBP) was performed on European–American individuals treated with metoprolol (*n* = 198) and chlorthalidone (*n* = 181), to test associations between 856 metabolites and baseline PRA. Metabolites with a false discovery rate (FDR) < 0.05 or *p* < 0.01 were tested for replication in 463 European–American individuals treated with atenolol or hydrochlorothiazide. Replicated metabolites were then tested for validation based on the directionality of association with BP response. Sixty-three metabolites were associated with baseline PRA, of which nine, including six lipids, were replicated. Of those replicated, two metabolites associated with higher baseline PRA were validated: caprate was associated with greater metoprolol SBP response (β = −1.7 ± 0.6, *p* = 0.006) and sphingosine-1-phosphate was associated with reduced hydrochlorothiazide SBP response (β = 7.6 ± 2.8, *p* = 0.007). These metabolites are clustered with metabolites involved in sphingolipid, phospholipid, and purine metabolic pathways. The identified metabolic signatures provide insights into the mechanisms underlying BP response.

## 1. Introduction

Hypertension (HTN) affects 46% of the US European American adults [1]. It is associated with an estimated annual cost of USD 52.4 billion and a mortality rate reaching 137.7 per 100,000 for European American men and 101.9 per 100,000 for European American women [1]. Three-quarters of European American hypertensive patients have uncontrolled blood pressure (BP) despite being prescribed antihypertensive medications [1,2]. Poor BP control is caused, in part, by a selection of antihypertensive therapy with mechanisms of action discordant from the pathophysiologic pathway(s) underlying HTN [3]. A personalized treatment approach, matching an individual’s hypertensive pathophysiologic pathway with an antihypertensive drug mechanism of action, may improve BP response and control [4].

The renin–angiotensin–aldosterone system (RAAS) is an important and highly variable BP regulator. The activity of this system can be assessed by measuring plasma renin activity (PRA). Previous studies demonstrate that PRA is a predictive biomarker of BP response to antihypertensive agents particularly in European American hypertensive patients [5,6,7,8]. Patients with lower PRA (< 0.65 ng/mL/h) achieve greater BP reductions in response to thiazide diuretics than β-blockers, whereas those with higher PRA (≥ 0.65 ng/mL/h) respond better to β-blockers than thiazide diuretics [9,10,11]. However, PRA, other clinical factors and the genetic variants identified to date, including those related to PRA, only explain about 3–4% of the total variability in the BP trait [12,13,14].

Metabolomics is an emerging tool that defines perturbations in metabolic pathways and informs the molecular basis for a phenotype [15,16]. The human metabolome is regulated by net interactions between genetic and environmental influences [17,18]. Several metabolic pathways have been linked to HTN and antihypertensive drug responses [19,20,21,22,23,24,25], but no evidence exists for metabolic signatures of PRA and the link to BP response. In the present study, we hypothesized that at least part of the variability in BP response among European American hypertensives is driven by metabolites related to PRA. Using an untargeted metabolomics approach, we aimed to identify the metabolites associated with pre-treatment PRA in European American hypertensive individuals, followed by replication in an independent cohort. We then sought to validate the successfully replicated metabolic signals with respect to BP responses to β-blockers and thiazide diuretics. We also aimed to identify common clusters of our validated metabolites, allowing us to further understand the metabolic processes underlying PRA and BP responses in European Americans.

## 2. Results

### 2.1. Study Population

The primary analysis of the current study included data from the principal component analysis (PCA)-determined European American participants enrolled in the Pharmacogenomic Evaluation of Antihypertensive Responses-2 (PEAR-2) trial (discovery cohort) and PEAR trial (replication cohort). We restricted the analysis to European American participants based on our previous findings that PRA is a predictive biomarker of BP response in Europeans, but not in African Americans [7,11]. A total of 198 metoprolol- and 181 chlorthalidone-treated PEAR-2 participants and a total of 463 PEAR participants treated with atenolol monotherapy or HCTZ monotherapy were included in this study (Figure S1). Baseline characteristics for the participants from both cohorts were similar (Table 1). Participants were 50 years old, on average and 44% were women; mean body mass index was 30.5 kg/m^2^ and median baseline PRA was 0.9 ng/mL/h.

### 2.2. Data Processing and Quality Control on PEAR-2 Metabolomics Data

Of the 1132 metabolites (761 structurally known and 371 unknown) detected in PEAR-2 samples, 276 were removed, including all xenobiotics (*n* = 165), metabolites with a constant or single value across samples (*n* = 13) and metabolites with >60% missing data (*n* = 98). The remaining 856 metabolites included in the quality control (QC) and final analyses consisted of 530 known and 326 unknown compounds. The PCA showed no clustering among the PEAR-2 participants. However, four outliers were identified (Figure S2). Eleven participants had outlying metabolic states and were flagged based on the pairwise standard Euclidean distance (SED) values. Five of those were found to have >10% missing metabolomics data. Of these five, three had extreme lipid (triglycerides, LDL and HDL) values (Table S2). The Bland–Altman (BA) method, which was used to assess the concordance of the metabolomics data between each pair, flagged 23 metabolites. Greater than 5% of the values for these metabolites were considered outliers (Table S3). The top 10% of metabolites with the largest coefficient of variation (CV) values (*n* = 37) are listed in Table S4. We included all the participants and metabolites flagged by the above QC steps. However, a sensitivity analysis was conducted, excluding the flagged participants and a further investigation of the flagged metabolites was performed if any of them were one of the top signals. More details on the data processing and QC results are illustrated in the Supplement.

### 2.3. Untargeted Metabolomics Analysis

**Figure 1:**
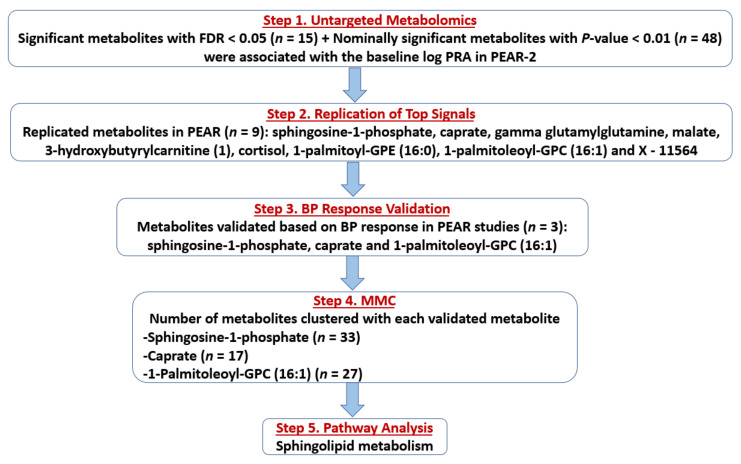
Flow chart showing the flow of the study results. Abbreviations: FDR, false discovery rate; PRA, plasma renin activity; PEAR, Pharmacogenomic Evaluation of Antihypertensive Responses; BP, blood pressure; MMC, Modulated Modularity Clustering.

The flow of the study results is summarized in Figure 1. Associations between baseline log-transformed levels (in terms of raw area counts) of each of the 856 PEAR-2 metabolites (non-imputed data) and baseline log-transformed PRA were assessed. Based on a false discovery rate (FDR) < 0.05, 15 metabolites were significantly associated with baseline log-transformed PRA (Step 1), including five lipids (four involved in sphingolipid metabolism), three amino acids, one energy metabolite and six structurally unknown metabolites. Twelve of these metabolites were associated with higher baseline PRA (Table 2). Additionally, 48 metabolites had nominal associations (suggestive *p* < 0.01) with baseline log-transformed PRA, including 22 lipids involved in fatty acid, phospholipid and glycerolipid metabolic pathways, among others. Of these 48 metabolites, 40 were associated with higher baseline PRA levels (Table S5).

Similar results were obtained after conducting a sensitivity analysis, excluding those participants with outlying baseline PRA (*n* = 13) and those flagged by PCA (*n* = 4) and SED (*n* = 11) QC steps.

### 2.4. Replication of Top Signals

Of the 63 total metabolites identified in Step 1 (significant plus nominally significant), 46 (8 metabolites with FDR < 0.05 + 39 metabolites with *p* < 0.01) had available data in the PEAR replication cohort (Table S6). One metabolite, X–21815, had > 60% missing data and was excluded. Associations between baseline log-transformed levels of each of the 46 metabolites (non-imputed data) moved to replication and baseline log-transformed PRA were assessed in PEAR. Of those 46 metabolites, 9 metabolites were significantly associated with baseline log-transformed PRA in the same direction as in PEAR-2 at an FDR < 0.05 (Step 2). Of those, eight metabolites were associated with higher PRA, including sphingosine-1-phosphate (a lipid involved in sphingolipid metabolism), caprate (a lipid, medium-chain fatty acid), 3-hydroxybutyrylcarnitine (1) (a lipid involved in fatty acid metabolism), cortisol (a lipid, steroid), 1-palmitoyl-GPE (16:0) and 1-palmitoleoyl-GPC (16:1) (two lysolipids), malate (an energy metabolite involved in tricarboxylic acid cycle (TCA)) and an unknown metabolite. In contrast, only gamma-glutamylglutamine (a peptide, gamma-glutamyl amino acid) was associated with lower baseline log-transformed PRA (Figure 1; Table 3). No successfully replicated metabolites were flagged by the BA or CV QC steps (Tables S3 and S4).

### 2.5. BP Response Validation

The nine successfully replicated metabolites were then tested for validation based on BP response in PEAR-2 and PEAR separately, which was assessed after ~8–9 weeks of treatment. Among those nine metabolites, three were associated with BP response (Step 3). Two (caprate and sphingosine-1-phosphate) that were associated with higher baseline PRA, were also significantly associated with BP responses to metoprolol and HCTZ, respectively, in the expected directions (validated). Specifically, the baseline levels of caprate were associated with a greater systolic BP (SBP) reduction to metoprolol (β = −1.7 ± 0.6, *p* = 0.006), which means that a 10% increase in the baseline levels of caprate was associated with about 0.2% greater SBP reduction to metoprolol. On the other hand, levels of sphingosine-1-phosphate were associated with reduced SBP and diastolic BP (DBP) responses to HCTZ (β = 7.6 ± 2.8, *p* = 0.007; β = 4.1 ± 1.7, *p* = 0.018, respectively), which means that a 10% increase in the baseline levels of sphingosine-1-phosphate was associated with about 0.7% and 0.4% lower reduction in SBP and DBP responses to HCTZ, respectively. Additionally, the metabolite 1-palmitoleoyl-GPC (16:1) associated with higher baseline PRA was nominally associated with reduced HCTZ SBP response (β = 4.1 ± 2.0, *p* = 0.038). This means that a 10% increase in the baseline levels of 1-palmitoleoyl-GPC (16:1) was associated with a 0.4% lower reduction in SBP to HCTZ (Figure 1; Table 4).

### 2.6. Modulated Modularity Clustering

The PEAR-2 metabolites that were clustered with each validated metabolite were identified using the Modulated Modularity Clustering (MMC), (Step 4). Caprate clustered with 17 metabolites, of which 6 were also lipids mainly involved in plasmalogen pathway. Sphingosine-1-phosphate clustered with 33 metabolites, of which 12 were also lipids (most involved in sphingolipid and phospholipid metabolism) and 7 were nucleotides involved in purine and pyrimidine metabolism. Lastly, the metabolite 1-palmitoleoyl-GPC (16:1) clustered with 27 metabolites, of which 16 were also lipids including lysolipids, monoacylglycerols and lipids involved in phospholipid metabolism. (Figure 1; Table S7).

### 2.7. Pathway Enrichment Analysis of Validated and Clustered Metabolites

**Figure 2:**
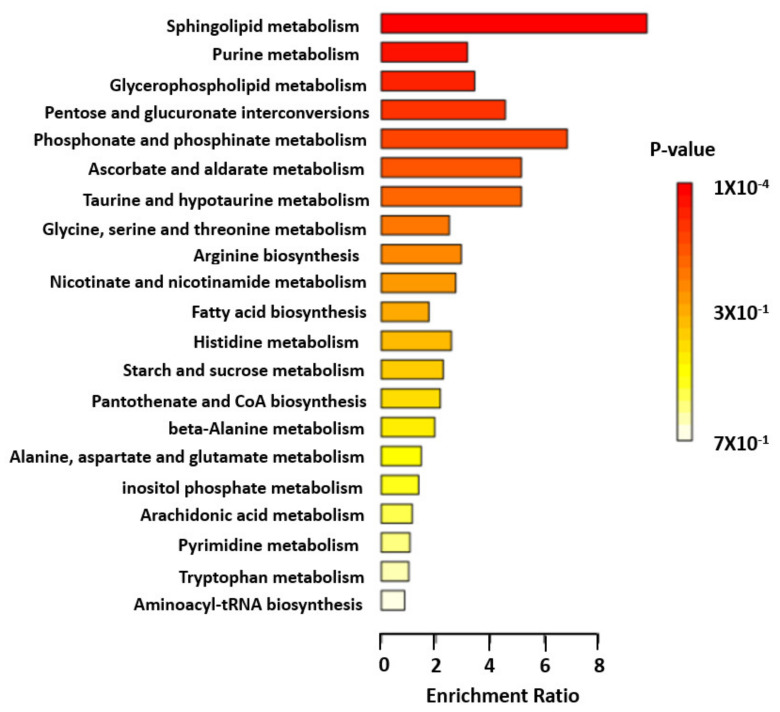
Pathway analysis of validated and clustered metabolite. Enrichment ratio of each top metabolic pathway is shown. Color indicates level of statistical significance, with darker red reflecting smaller *p*-values and lighter color down to white reflecting larger *p*-values.

Of the 64 validated and clustered metabolites, 41 had human metabolome database (HMDB) identifiers, which were imported into the MetaboAnalyst 5.0 enrichment analysis function [26]. The pathway analysis identified sphingolipid metabolic pathway as the only significantly impacted pathway (enrichment ratio, 9.8; FDR = 0.0083) (Step 5). Other top pathways included purine, glycerophospholipid, pentose and phosphonate metabolic pathways (Figure 2; Table S8).

## 3. Discussion

To our knowledge, this is the first pharmacometabolomic study to identify PRA metabolomic signatures and link them with BP response to antihypertensive therapy. Using a stepwise approach, we identified and replicated nine metabolic signals (six lipids, an energy metabolite, a peptide and an unknown metabolite) that were associated with baseline PRA among two cohorts of European American participants with uncomplicated HTN. Of those replicated, two (caprate and sphingosine-1-phosphate) were validated based on BP responses to metoprolol and HCTZ, respectively. Moreover, we found that these validated metabolites were clustered with several metabolites involved in sphingolipid, phospholipid, plasmalogen, purine and leucine, isoleucine and valine metabolic pathways, highlighting these as potential pathways underlying PRA and BP response.

Our analysis showed that the medium-chain fatty acid caprate (10:0) was associated with higher baseline PRA levels in PEAR-2 and that this association was successfully replicated in the PEAR cohort. We also showed that caprate was associated with a better SBP response to metoprolol. Similar to our findings, Gleeson et al. reported that caprate (10:0) enhances the cellular penetration of the tripeptides, IIe-Pro-Pro (IPP) and Leu-Lys-Pro (LKP) which inhibit the angiotensin-converting enzyme (ACE) (a component of the RAAS) and result in BP reduction in isolated rat jejunal tissue [27]. Although none of the human metabolomic studies published to the date indicated that caprate (10:0) is a potential biomarker of any of the HTN-related phenotypes, our study demonstrated that it was clustered with several plasmalogens. Increased levels of plasmalogens have been associated with increased oxidative stress and with higher cardiovascular mortality in patients with end-stage renal disease [28]. These data suggest that plasmalogens might also be involved in the pathways underlying BP response. Of note, we are the first to our knowledge to identify a relationship between caprate and renin.

We also demonstrated that sphingosine-1-phosphate (a lipid involved in sphingolipid metabolism) was associated with higher baseline PRA in both discovery and replication cohorts and with reduced HCTZ BP response. Sphingosine-1-phosphate is a highly bioactive lipid linked to HTN pathophysiology and elevated BP through vasoconstriction in animal studies [29,30,31]. A study suggested that the sphingosine-1-phosphate induced vasoconstriction is mediated through activation of the RhoA signaling cascade [32]. In contrast, it has been demonstrated that one of the mechanisms by which HCTZ chronically lowers BP is through a reduction in the RhoA and Rho kinase expression in the vascular smooth muscle cells shown in vitro in a previous study, which leads to direct vasodilation [33,34]. This hypothesized mechanism might explain the relationship shown in this study between sphingosine-1-phosphate and reduced HCTZ response.

We also recently found an association between N24:2 sphingomyelin and better HCTZ BP response [25]. We additionally showed that European American carriers of the C-allele of the genetic variant rs6078905 within the *SPTLC3* gene, which is associated with higher levels of N24:2 sphingomyelin had a better HCTZ BP response compared to non-carriers [25]. The reasons behind the observed opposite effects of sphingosine-1-phosphate and N24:2 sphingomyelin on HCTZ response are not well understood. However, each of these metabolites is synthesized through a different pathway which might affect BP in a different way: shingosine-1-phsophate is synthesized by degradation of sphingolipids in the plasma membrane or lysosomal compartment, whereas sphingomyelin is synthesized from ceramide in Golgi apparatus [35]. Moreover, in the present study, sphingosine-1-phosphate was clustered with several metabolites implicated in sphingolipid, phospholipid and purine metabolism, which have been previously associated with HTN, BP and antihypertensive responses [20,36,37,38]. Additionally, from the pathway analysis, we found that sphingolipid metabolic pathway was the only significantly impacted pathway, which emphasizes the importance of this pathway for BP response.

In addition to its link with HTN and BP response, sphingosine-1-phosphate has also been shown to alleviate congestive heart failure induced by increased cardiac renin release [39]. Angiotensin II (a component of the RAAS) activates the Rho-kinase pathway, which is also activated by sphingosine-1-phosphate [32,40]. Together, these data suggest that the sphingolipid metabolism pathway might be involved in the underlying antihypertensive responses through the RhoA-kinase pathway and possibly through the RAAS pathway. Further work on sphingolipid metabolic pathway might provide more insights particularly on the link to the RAAS pathway.

We also found that 1-palmitoleoyl-GPC (16:1) was associated with higher baseline PRA in our discovery and replication cohorts and was nominally associated with a reduced SBP response to HCTZ. Consistent with our findings, previous studies indicated that palmitic amide and palmitic acid (structurally related metabolites) were found to be associated with HTN [36,41,42]. Additionally, 1-palmitoleoyl-GPC (16:1) was clustered with several lipids involved in phospholipid metabolic pathway, which have been previously associated with HTN [36,37,41].

Our study has several strengths. The results of the present study were obtained through a stepwise approach, including replication of our findings and further validation which confirms the significance of our results and the influence on PRA phenotype and on BP response complex phenotype. Additionally, the use of MMC clustering and of the pathway enrichment analysis helped us narrow and highlight the most biologically relevant metabolic pathways underlying PRA and BP responses. Our study has also several limitations. One limitation is the unavailability of several PEAR-2 metabolic signals in the PEAR replication dataset which might have resulted in missing other potential metabolic biomarkers of PRA and BP response. Additionally, none of our successfully replicated metabolites were associated with BP responses to both the β-blockers and the diuretics used in the PEAR studies, in the expected directions. This might be because BP response is a complex trait, affected by many other genetic and environmental factors, not related to the RAAS. Additionally, our validation phase was limited to BP response to β-blockers and thiazide diuretics. Future studies are warranted to test the association between the PRA-related metabolites and BP response to other RAAS blockers such as ACE inhibitors and angiotensin II receptor blockers (ARBs). Moreover, the relatively small sample size in our study might have limited our power to detect additional metabolic signals associated with PRA and BP responses.

## 4. Materials and Methods

### 4.1. Study Design and Participants

Both PEAR studies were conducted in accordance with the Declaration of Helsinki and the protocols were approved by the institutional review boards at all participating sites (University of Florida in Gainesville, FL, USA; Mayo Clinic in Rochester, MN, USA; and Emory University in Atlanta, GA, USA). All participants provided voluntary, written informed consent prior to participation. In both studies, participants recruited had either treated or untreated uncomplicated primary HTN. For the treated participants, all antihypertensive drugs (including β-blockers, calcium channel blockers, ACE inhibitors, ARBs, diuretics and α-blockers) were discontinued prior to treatment with the study drug, with a washout period of 4–6 weeks, to allow for re-establishment of their hypertensive status. In both studies, pre- and post-treatment BPs were measured, and blood samples were collected at baseline to measure PRA and to conduct the global metabolomics analysis. Both PEAR trials were conducted to assess ‘omics responses to the commonly prescribed antihypertensive drug classes including thiazide diuretics and β-blockers. Currently, thiazide diuretics are first-line antihypertensive agents [43]. Although β-blockers are now reserved for hypertensive patients requiring multiple antihypertensive classes for BP control, about 20% of US adults with uncomplicated HTN are still prescribed β-blockers [43,44]. Further details about study designs are described below and in the Supplementary Materials.

The PEAR-2 and PEAR trials have been previously described in detail [45,46]. Briefly, PEAR-2 was a prospective, multicenter, open-label, sequential clinical trial (clinicaltrials.gov identifier: NCT01203852). Study participants with uncomplicated HTN aged 18–65 years old were sequentially treated with the β-blocker metoprolol monotherapy, followed by the thiazide-like diuretic chlorthalidone monotherapy with an intervening 4-week washout. Participants with cardiovascular disease, diabetes mellitus, renal or hepatic dysfunction were excluded from enrollment.

PEAR was a prospective, multicenter, randomized, open-label, crossover clinical trial (clinicaltrials.gov identifier: NCT00246519) and included a similar population to the one in PEAR-2 with the same exclusion criteria. Participants were randomly assigned to either atenolol (β-blocker) followed by hydrochlorothiazide (HCTZ) (thiazide diuretic) as add-on therapy, or HCTZ followed by atenolol (as add-on therapy).

### 4.2. BP Phenotype

In both PEAR studies, clinic and home BPs at baseline (pre-treatment) and post-treatment were measured (post-treatment ~8 weeks in PEAR-2 and ~9 weeks in PEAR and occurred after dose titrations). In the PEAR-2 study, clinic and home BPs were measured using monitors manufactured by Microlife, model #s BP3AC1-PC and BP3MC1-PC (Dunedin, FL, USA), which were set to obtain three sequential BP measurements separated by two minutes each, and the average of the three measurements was used [45]. In the PEAR study, clinic and home BPs were also taken in a triplicate mode using an automated oscillometric sphygmomanometer (Microlife 3AC1-PC; Microlife, Minneapolis, MN, USA), and the average of the three values was used [46]. Clinic BP measurements were used in the analyses of the current study since most treatment decisions are based on clinic BP [47].

### 4.3. PRA Determination

Baseline PRA in both cohorts was measured in plasma samples collected before treatment [11,46]. Samples were collected under the study participant’s normal sodium intake and usual clinical settings based on the evidence that PRA measurement is not significantly affected by sodium restriction and does not need 24 h urinary collection [48]. To prevent cryoactivation of prorenin to renin, blood samples were processed at room temperature, then stored frozen. The standard incubation time was 3 h but was extended to 18 h in samples with PRA < 1 ng/mL/h. PRA was measured at pH 5.7 in all samples in duplicate or triplicate by radioimmunoassay of the generated angiotensin-I according to Sealy’s method, and the mean value for each participant was used in analyses [49]. The assay was conducted in the central laboratory at Mayo Clinic, Rochester, MN, USA using reagents purchased from the DiaSorin company (Stillwater, MN, USA). PRA was reported as the amount of angiotensin-I generated (in ng/mL) per hour [11,46].

### 4.4. Untargeted Metabolomics Profiling

Baseline fasting plasma samples from PEAR-2 and PEAR participants were used for the metabolomics analysis. Untargeted/global metabolite profiling was conducted by Metabolon using ultrahigh performance liquid chromatography-tandem mass spectroscopy (UPLC-MS/MS) (Waters, Milford, MA, USA) [50]. Samples were divided into aliquots and stored at −80 °C until processed. At the time of the analysis, an aliquot was thawed and extracted with methanol and then centrifuged to remove proteins and recover the metabolites. The resulting extract was divided into five aliquots. Two aliquots were analyzed by two separate reverse phases (RP)/UPLC-MS/MS with a positive ion mode electrospray ionization (ESI); one was chromatographically optimized for more hydrophilic compounds, and the other was optimized for more hydrophobic compounds. In this method, the extracts were gradient eluted from a C18 column. Another aliquot was analyzed by RP/UPLC-MS/MS with a negative ion mode ESI using a separate dedicated C18 column. The fourth aliquot was analyzed by hydrophilic interaction liquid chromatography (HILIC)/UPLC-MS/MS with a negative ion mode ESI. The fifth aliquot was reserved for backup. All methods utilized a Waters ACQUITY UPLC and a Thermo Scientific Q-Exactive high resolution/accurate mass spectrometer interfaced with a heated electrospray ionization (HESI-II) source and Orbitrap mass analyzer operated at 35,000 mass resolution (Waters, Milford, MA, USA). Compounds were identified by comparison to library entries of purified standards or recurrent unknown entries. For each metabolite, peaks were quantified using the area under the curve in each sample. More details on metabolomics profiling are presented in the Supplementary Materials.

### 4.5. Data Processing and QC on PEAR-2 Metabolomics Data

MetaboAnalyst 5.0, an open-source R-based program for metabolomics, and Galaxy SECIM tools were used to perform processing and QC on the PEAR-2 metabolomics data [26,51]. Details on this work are presented in the Supplementary Materials. Briefly, all the xenobiotics, metabolites with a constant or single value across samples and metabolites with greater than 60% missing data were excluded. The remaining metabolites (non-imputed) were included in the QC steps and in the analysis. The K-nearest neighbors (KNN) algorithm was used to impute the data only to perform the PCA, which was performed to identify if there are any clustering or extreme outliers among the participants based on their metabolomics data. Participants with outlying metabolic states were identified using the pairwise SED. The concordance of the metabolomics data between each pair within each subgroup was assessed using BA method, with the expectation that participants within the same subgroup have similar metabolic states [52]. Additionally, CV was calculated for each of the remaining metabolites to assess their variability across participants.

### 4.6. Statistical Analyses

Characteristics of the PEAR-2 and PEAR participants were analyzed using descriptive statistics. Data for continuous variables are presented as means with standard deviations (SDs), except for PRA, which is not normally distributed, thus is presented as median with inter-quartile range (IQR). Data for categorical variables are presented as numbers and percentages. In the PEAR-2 discovery cohort, baseline (pre-metoprolol and pre-chlorthalidone) PRA outliers were determined based on standardized residuals of greater than three SDs, Cook’s distance, and DFFITS. Participants with outlying PRA values (*n* = 13) were included in the analyses because they were also considered physiological outliers as they had extreme BP and metabolic responses. Metabolomics data in both cohorts were also not normally distributed. Therefore, log-transformed PRA and log-transformed metabolomics data (non-imputed) were used in all analyses. BP responses in both cohorts were defined by subtracting the pre-treatment BP from the post-treatment BP, such that negative values were indicative of BP reductions. In this study, we utilized a 5-step analytic approach as outlined below. All statistical analyses were carried out with SAS (version 9.4; SAS Institute Inc., Cary, NC, USA) and R Statistical Software (Foundation for Statistical Computing, Vienna, Austria).

#### 4.6.1. Step 1: Untargeted Metabolomics Analysis

After data processing, associations between baseline levels (in terms of raw area counts) of each of the PEAR-2 metabolites included in the analysis and baseline PRA were assessed using multivariable linear regression models, adjusted for age, sex and baseline SBP. These variables were selected based on their significant associations with baseline PRA in PEAR-2. Missing values for each tested metabolite were ignored, resulting in varying sample sizes for each metabolite association. We used two different approaches to identify the metabolic signals associated with PRA: a strict approach by selecting the significant metabolites with an FDR < 0.05, and a broader approach by selecting the nominally significant metabolites with a suggestive *p* < 0.01.

Also, a sensitivity analysis was conducted, excluding those participants with outlying baseline PRA and those participants flagged by the PCA and SED QC steps. Metabolites that were flagged by BA and CV were included in the analysis but planned to be further investigated if any of them were one of the top signals.

#### 4.6.2. Step 2: Replication of Top Signal(s)

Metabolic signals identified based on the above pre-specified thresholds were tested for replication by assessing the association of each with baseline PRA in an independent cohort (PEAR European Americans). Replication was performed using multivariable linear regression, adjusted for age, sex and baseline SBP. Metabolites were considered successfully replicated if they had the same direction of effect to the PEAR-2 metabolomic findings and had an FDR < 0.05.

#### 4.6.3. Step 3: BP Response Validation

Given the well-documented relationship between PRA and BP response to anti-renin drugs (including β-blockers) and thiazide diuretics [6,9,10], we hypothesized that metabolites associated with higher PRA would be associated with better BP response to metoprolol and atenolol and poorer BP responses to chlorthalidone and HCTZ (thiazide diuretics), and vice versa for those metabolites associated with lower PRA. To test this hypothesis, we assessed the direction of association of each successfully replicated metabolite, with BP response, in the PEAR-2 and PEAR studies separately using multivariable linear regression, adjusted for age, sex and baseline SBP and DBP. For a metabolite to be considered validated, it should be associated with BP response to at least one of the four drugs in the expected direction at a *p* < 0.0125 (0.05/4). Nominal validation is considered at a suggestive *p* < 0.05.

#### 4.6.4. Step 4: MMC

We then used the MMC tool in Galaxy SECIM tools to identify the PEAR-2 metabolites clustered with each validated metabolite. This was performed using pairwise Pearson correlations [53].

#### 4.6.5. Step 5: Pathway Enrichment Analysis of Validated and Clustered Metabolites

Lastly, the HMDB identifiers of the validated and the clustered metabolites were imported into the MetaboAnalyst 5.0 enrichment analysis function, which investigated if a group of functionally related metabolites is significantly enriched. The enrichment ratio per metabolite set was computed by dividing the observed by the expected hits. The significance threshold was set at FDR < 0.05. The pathway library included 84 metabolite sets based on the Kyoto Encyclopedia of Genes and Genomes (KEGG) human metabolic pathways [26].

## 5. Conclusions

In conclusion, our study reveals several metabolic pathways that might be involved in the mechanisms underlying the relationship between RAAS activity and BP responses to β-blockers and thiazide diuretics in European–American patients with uncomplicated HTN. Personalizing antihypertensive therapy based on an individual’s hypertensive pathophysiology has long been the holy grail of clinicians. As data such as ours are confirmed and omics testing becomes clinically available, it will be possible to select antihypertensive medications based on biomarkers suggesting optimal responses with the potential for improved BP control. Additional research on the identified and clustered metabolites as well as integrating them with other omics data may provide us with more insights into the mechanisms underlying BP-lowering effects of β-blockers and thiazide diuretics. This may also open avenues for developing new antihypertensive agents targeting specific metabolic pathways.

## Figures and Tables

**Table 1:** Characteristics of the European American participants included in PEAR-2 and PEAR studies.

Baseline Characteristics				
Variable	PEAR-2		PEAR	
	Metoprolol (*n* = 198)	Chlorthalidone (*n* = 181)	Atenolol (*n* = 233)	HCTZ (*n* = 230)
Age, years	51.1 ± 9	51.1 ± 8.9	49.6 ± 9.5	50 ± 9.4
Females, N (%)	89 (45%)	77 (42.5%)	109 (46.8%)	93 (40.4%)
BMI, kg/m^2^	30.8 ± 5.1	30.6 ± 5	30.3 ± 5.6	30.3 ± 4.9
Baseline PRA, ng/mL/h	0.91 (0.49–1.77)	0.85 (0.55–1.46)	0.9 (0.46–1.52)	0.87 (0.46–1.43)
Baseline SBP, mmHg	150 ± 12.2	151.2 ± 13.2	151.1 ± 12.4	151.8 ± 12.5
Baseline DBP, mmHg	97.8 ± 5.2	98.9 ± 5.5	97.9 ± 5.7	98.1 ± 5.8
**Post-treatment BP**				
Post-treatment SBP, mmHg	136.8 ± 15.4	136 ± 12.7	135.7 ± 14.6	140.8 ± 13.5
Post-treatment DBP, mmHg	86.7 ± 8.3	90.3 ± 8.2	85.9 ± 8.9	93.1 ± 8.3
SBP response *, mmHg	−13.2 ± 13.7	−15.3 ± 13.9	−15.4 ± 14.7	−11 ± 12.8
DBP response *, mmHg	−11.1 ± 8.2	−8.6 ± 8.3	−12 ± 9	−5 ± 7.2

All continuous variables are presented as means with standard deviations (SD), except for PRA, which was not normally distributed, thus presented as median with interquartile range (IQR). * BP response calculated as post-treatment BP minus pre-treatment BP. Abbreviations: PEAR, Pharmacogenomic Evaluation of Antihypertensive Responses; HCTZ, hydrochlorothiazide; BMI, body mass index; PRA, plasma renin activity; SBP, systolic blood pressure; DBP, diastolic blood pressure.

**Table 2:** The fifteen metabolites significantly associated with baseline log-transformed PRA in PEAR-2 European Americans.

Metabolite Name	Classification	Pathway	HMBD	Estimate ± SE	*p*-Value	FDR
Sphinganine-1-phosphate	Lipid	Sphingolipid Metabolism	HMDB01383	0.21 ± 0.05	4.45 × 10^−5^	0.01
Sphingomyelin (d18:1/20:1, d18:2/20:0)	Lipid	Sphingolipid Metabolism	unknown	0.09 ± 0.02	0.00012	0.026
Sphingosine-1-phosphate	Lipid	Sphingolipid Metabolism	HMDB00277	0.24 ± 0.06	0.0002	0.033
Sphinganine	Lipid	Sphingolipid Metabolism	HMDB00269	0.09 ± 0.03	0.0007	0.042
Caprate (10:0)	Lipid	Medium Chain Fatty Acid	HMDB00511	0.04 ± 0.01	0.0005	0.042
N-acetylglutamate	Amino Acid	Glutamate Metabolism	HMDB01138	0.26 ± 0.07	0.00018	0.03
Beta-hydroxyisovalerate	Amino Acid	Leucine, Isoleucine and Valine Metabolism	HMDB00754	0.14 ± 0.04	0.0007	0.042
Threonine	Amino Acid	Glycine, Serine and Threonine Metabolism	HMDB00167	0.11 ± 0.03	0.0008	0.045
Fumarate	Energy Metabolite	TCA Cycle	HMDB00134	0.22 ± 0.06	0.0007	0.042
3-Hydroxybutyroylglycine	Lipid	Fatty Acid Metabolism	NA	0.21 ± 0.05	1.57 × 10^−5^	0.007
3-Hydroxystachydrine	Unknown	Unknown	NA	0.1 ± 0.03	0.0006	0.042
1-Methyl-5-imidazoleacetate	Unknown	Unknown	NA	−0.94 ± 0.27	0.0006	0.042
Glucuronide of C10H18O2 (7)	Unknown	Unknown	NA	−0.08 ± 0.02	0.0007	0.042
X–12726	Unknown	Unknown	NA	0.23 ± 0.05	1.45 × 10^−5^	0.007
X–12818	Unknown	Unknown	NA	−0.11 ± 0.03	0.0003	0.042

*p*-values were produced using linear regression analysis of each metabolite with the baseline log-transformed plasma renin activity (PRA) in PEAR 2 European Americans, with adjustment of age, sex and baseline systolic blood pressure (SBP). False discovery rate (FDR) with a significant threshold of less than 0.05 was used to account for multiple comparisons. Abbreviations: PRA, plasma renin activity; PEAR, Pharmacogenomic Evaluation of Antihypertensive Responses; SE, standard error; TCA, tricarboxylic acid cycle; HMDB, Human Metabolome Database; FDR, false discovery rate; NA, not applicable.

**Table 3:** Replicated metabolites in PEAR European Americans.

Metabolite Name	Estimate ± SE	*p*-Value	FDR
Sphingosine-1-phosphate	0.19 ± 0.06	0.002	0.02
Caprate (10:0)	0.18 ± 0.05	0.0002	0.003
Gamma-glutamylglutamine	−0.11 ± 0.03	6.59 × 10^−5^	0.002
Malate	0.21 ± 0.06	0.0009	0.01
3-Hydroxybutyrylcarnitine (1)	0.10 ± 0.02	3.99 × 10^−6^	0.0001
Cortisol	0.11 ± 0.04	0.003	0.02
1-Palmitoyl-GPE (16:0)	0.12 ± 0.04	0.004	0.02
1-Palmitoleoyl-GPC (16:1) *	0.13 ± 0.04	0.002	0.02
X–11564	0.21 ± 0.07	0.004	0.02

These results were generated using linear regression models of each metabolite with the baseline log-transformed plasma renin activity (PRA) in PEAR European Americans, with adjustment for age, sex and baseline systolic blood pressure (SBP). False discovery rate (FDR) with a significant threshold of less than 0.05 was used. Abbreviations: PEAR, Pharmacogenomic Evaluation of Antihypertensive Responses; SE, standard error; FDR, false discovery rate.

**Table 4:** Validated metabolites and replicated metabolites with nominal associations with BP responses in the expected direction in PEAR studies.

Metabolite	Metoprolol SBP Response		Metoprolol DBP Response	
	Estimate ± SE	*p*-Value	Estimate ± SE	*p*-Value
Caprate	−1.7 ± 0.6	0.006	−0.7 ± 0.4	0.05
	**HCTZ SBP response**		**HCTZ DBP response**	
	**Estimate ± SE**	***p*-value**	**Estimate ± SE**	***p*-value**
Sphingosine-1-phosphate	7.6 ± 2.8	0.007	4.1 ± 1.7	0.018
1-Palmitoleoyl-GPC (16:1) *	4.1 ± 2.0	0.038	1.1 ± 1.2	0.3

These results were generated using linear regression models of each metabolite with the SBP and DBP responses to metoprolol, atenolol and HCTZ in PEAR-2 and PEAR European Americans, with adjustment for age, sex and baseline SBP/DBP. Caprate and sphingosine-1-phosphate were considered validated (*p* < 0.0125), while 1-palmitoleoyl-GPC (16:1) * was nominally validated (*p* < 0.05). Abbreviations: BP, blood pressure; PEAR, Pharmacogenomic Evaluation of Antihypertensive Responses; SBP, systolic blood pressure; DBP, diastolic blood pressure; HCTZ, hydrochlorothiazide.

## Data Availability

The data presented in this study are available on request from the corresponding author. The data are not publicly available due to ethical and privacy concerns.

## Supplementary Materials

The following are available online at https://www.mdpi.com/article/10.3390/metabo11090645/s1, Figure S1: Consort diagram showing the participants included in this study, Figure S2: PCA scatter plots showing clustering of the PEAR-2 samples (*n* = 379) based on the first three PCs, Table S1: Percent of variability in metabolomics data explained by each one of the first 10 PCs, Table S2: The 11 PEAR-2 participants with the largest SEDs, Table S3: The 23 metabolites flagged by BA plots and measures, Table S4: The top 10% of the metabolites with the largest CV values (*n* = 37), Table S5: The 48 metabolites nominally associated with the baseline log PRA in PEAR-2 European Americans with *p* < 0.01, Table S6: Availability of the metabolites having significant or nominally significant associations with the baseline PRA from discovery phase (*n* = 63) in PEAR, Table S7: The metabolites clustered with caprate, sphingosine-1-phosphate and 1-palmitoleoyl-GPC (16:1) *, Table S8: List of top metabolic pathways enriched in the pathway analysis.
