# Supplementary material for: Metabolomics Signature of Plasma Renin Activity and Linkage with Blood Pressure Response to Beta Blockers and Thiazide Diuretics in Hypertensive European American Patients
Source: Metabolites. 2021 Sep 21;11(9):645. doi: 10.3390/metabo11090645 (PMC8466669; doi:10.3390/metabo11090645)
Supplement: Supplementary file 1 [file metabolites-11-00645-s001.zip › metabolites-1369855-supplementary.pdf]

metabolites

MDPI

Data Supplement

Metabolomics Signature of Plasma Renin Activity and Linkage with Blood Pressure Response to Beta Blockers and Thiazide Diuretics in Hypertensive European American Patients

Mai Mehanna[1], Caitrin W McDonough[1], Steven M Smith[1], Yan Gong[1], John G. Gums[1], Arlene B. Chapman[2], Julie A. Johnson[1], Lauren McIntyre[3] and Rhonda M Cooper-DeHoff[1]

[1]Department of Pharmacotherapy and Translational Research and Center for Pharmacogenomics, College of Pharmacy, University of Florida, Gainesville, Florida
[2]Department of Medicine, University of Chicago, Chicago, Illinois
[3]Department of Molecular Genetics and Microbiology, College of Medicine, University of Florida, Gainesville, Florida
* Correspondence: **Author:** Rhonda M. Cooper-DeHoff, PharmD, MS, Associate ProfessorDepartment of Pharmacotherapy and Translational ResearchColleges of Pharmacy and MedicineAssociate Director, Center for PharmacogenomicsUniversity of FloridaP.O. Box 100486Gainesville, FL 32610-0486Tel: (352) 273-6184Email: dehoff@cop.ufl.edu

Running Head: Metabolomics of PRA

Key Words: plasma renin activity; metabolomics; hypertension; blood pressure.

Trials' Registry Numbers: NCT01203852; NCT00246519

Methods:

Study design and participants in detail:

The Pharmacogenomics Evaluation of Antihypertensive Responses-2 (PEAR-2):

The primary analysis included PEAR-2 European American participants with baseline metabolomics data (discovery cohort). The PEAR-2 was a prospective, multicenter, open-label, sequential clinical trial, conducted at 3 sites (University of Florida in Gainesville, FL, Mayo Clinic in Rochester, MN, and Emory University in Atlanta, GA) (clinicaltrials.gov identifier: NCT01203852). Details on the PEAR-2 clinical trial have been previously published [1]. Study participants with uncomplicated mild to moderate essential hypertension (HTN), aged 18-65 years old, of any race were recruited. After an average washout period of 4 weeks of their current antihypertensive medications, participants were initially treated with the β-blocker metoprolol 50 mg twice daily for two weeks, followed by a dose titration to 100 mg twice daily for an additional six weeks. After a second washout period, the participants were treated with the thiazide-like diuretic chlorthalidone 15 mg once daily, followed by a dose titration to 25 mg once daily for a total of 6-8 weeks of treatment. Exclusion criteria were secondary HTN, systolic blood pressure (SBP) > 180 mmHg or diastolic BP (DBP) > 110 mmHg, isolated systolic HTN, cardiovascular disease, diabetes mellitus, heart rate < 55 beats/ min, renal or hepatic dysfunction. Also, pregnant and lactating women were excluded.

PEAR:

We used data from European American participants who received monotherapy in PEAR in the replication analysis. The PEAR was a prospective, multicenter, randomized, open-label, crossover clinical trial, conducted at the same 3 centers mentioned above (clinicaltrials.gov identifier: NCT00246519). The details of this study have been previously reported [2]. Participants with uncomplicated mild to moderate essential HTN, aged 17-65 years old, of any race were enrolled. After a washout period of about 4 weeks of any antihypertensives, participants were randomized to either the β-blocker atenolol 50 mg once daily (dose titrated to 100 mg once daily if BP remained above 120/70 mmHg) or the thiazide diuretic hydrochlorothiazide (HCTZ) 12.5 mg once daily (dose titrated to 25 mg once daily if BP remained above 120/70 mmHg) for a total of 9 weeks. If the BP remained above the goal after monotherapy treatment, drug from the other treatment arm was added (i.e. HCTZ for those on atenolol, and vice versa), followed by the same dose titration for another 6 to 9 weeks of treatment. PEAR exclusion criteria were the same as described above for the PEAR-2 study.

Untargeted metabolomics profiling in detail:

Baseline fasting plasma samples from PEAR-2 and PEAR participants were used for the untargeted metabolomics profiling conducted by Metabolon [3]. Following receipt, samples were stored at -80˚C until processed. Samples were then prepared by removing proteins and recovering the metabolites using methanol under vigorous shaking, followed by centrifugation. The resulting extract was divided into five aliquots: two for analysis by two separate reverse phase (RP)/ultra-performance liquid chromatography – mass spectrometry (UPLC-MS)/MS methods with positive ion mode electrospray ionization (ESI), one for analysis by RP/UPLC-MS/MS with negative ion mode ESI, one for analysis by hydrophilic interaction liquid chromatography (HILIC)/UPLC-MS/MS with negative ion model ESI, and one aliquot was reserved for backup. The sample extracts were stored overnight under nitrogen before preparation for analysis. Several types of controls were analyzed along with the experimental samples: a pooled matrix samples generated by taking a small volume of each experimental sample (or alternatively, use of a pool of well-characterized human plasma) served as a technical replicate, extracted water samples served as process blanks, and a cocktail of quality control (QC) standards that were carefully chosen not to interfere with the measurement of endogenous compounds were

spiked into each sample. Instrument variability was determined by calculating the median relative standard deviation (RSD) for the standards that were added to each sample before injection into the mass spectrometers. Overall process variability was determined by calculating the median RSD for all endogenous metabolites present in 100% of the pooled matrix samples. Samples were randomized across the platform run with QC samples placed evenly among the injections. All methods utilized a Waters ACQUITY UPLC and a Thermo Scientific Q-Extractive high resolution/ accurate mass spectrometer interfaced with a heated electrospray ionization (HESI-II) source and Orbitrap mass analyzer operated at 35,000 mass resolution. The sample extract was dried then reconstituted in solvents compatible to each of the four methods. One aliquot was analyzed using acidic positive ion conditions, chromatographically optimized for more hydrophilic compounds. In this method, the extract was gradient eluted from a C18 column (Water UPLC BEH C18-2.1x100 mm, 1.7 µm) using water and methanol, containing 0.05% perfluoropentanoic acid (PFPA) and 0.1% formic acid (FA). Another aliquot was also analyzed using acidic positive ion conditions but was chromatographically optimized for more hydrophobic compounds. In this method, the extract was gradient eluted from the same afore mentioned C18 column using methanol, acetonitrile, water, 0.05% PFPA and 0.01% FA and was operated at an overall higher organic content. Another aliquot was analyzed using basic negative ion optimized conditions using a separate C18 column. The basic extracts were gradient eluted from the column using methanol and water, but with 6.5 mM Ammonium Bicarbonate at pH 8. The fourth aliquot was analyzed using negative ionization following elution from a HILIC column (Waters UPLC BEH Amide 2.1x150 mm, 1.7 µm) using a gradient consisting of water and acetonitrile with 10 mM Ammonium Formate, pH 10.8. The scan range varied slightly between methods but covered 70-1000 m/z. Raw data files are archived, extracted, peak-identified and QC processed using Metabolon's hardware and software. Compounds were identified by comparison to library entries of purified standards or recurrent unknown entities. Metabolon maintains a library based on authenticated standards that contains the retention time/index (RI), mass to charge ration (*m*/*z*), and chromatographic data (including MS/MS spectral data) on all molecules present in the library. Also, biochemical identifications were based on three criteria: retention index within a narrow RI window of the proposed identification, accurate mass match to the library +/- 10 ppm, and the MS/MS forward and reverse scores between the experimental data and authentic standards. The MS/MS scores are based on a comparison of the ions present in the experimental spectrum to the ions present in the library spectrum. While there may be similarities between these molecules based on one of these factors, the use of all three data points can be utilized to distinguish and differentiate biochemicals. More than 3300 commercially available purified standard compounds have been acquired and registered for determination of their analytical characteristics. Additional mass spectral entries have been created for structurally unnamed biochemicals, which have been identified by virtue of their recurrent nature (both chromatographic and mass spectral). The QC and curation processes were designed to ensure accurate and consistent identification of true chemical entities, and to remove those representing system artifacts, mis-assignments, and background noise. Library matches for each compound were checked for each sample and corrected if necessary. Peaks were quantified using area-under-the-curve. A data normalization step was performed to correct variation resulting from instrument inter-day tuning differences. Each compound was corrected in run-day blocks by registering the medians to equal one (1.00) and normalizing each data point proportionately.

QC on PEAR-2 metabolomics data:

A total of 1132 metabolites have been detected using Metabolon platform in PEAR-2 plasma samples. These metabolites included 761 known/ named biochemicals (295 lipids, 179 amino acids, 165 xenobiotics, 35 nucleotides, 28 cofactors and vitamins, 27 peptides, 23 carbohydrates and 9 energy metabolites) and 371 unknown/ unnamed biochemicals. MetaboAnalyst 3.0, an open-source R-based program for metabolomics and Galaxy

SECIM tools were used to perform data processing and QC on the PEAR-2 baseline metabolomics data [4,5]. These steps are described in detail below. The purpose of QC was to detect and flag any outlying metabolite or sample which need further investigation to assess whether these are true values or are due to any technical or experimental error.

Data processing:

After data processing, a total of 276 metabolites were removed. First, all the xenobiotics (n=165) were excluded from the analysis to reduce the environmental confounding effects on our analysis. These included drugs' metabolites (n=68), food or plant metabolites (n=39), metabolites involved in benzoate metabolism (n=19), chemicals (n=19), metabolites involved in xanthine metabolism (n=15), tobacco metabolites (n=4) and bacterial/ fungal metabolite (n=1). Also, metabolites with a constant or single value across samples (n=13) were excluded since their effects on the outcome of interest may be ignored. Additionally, a total of 98 metabolites were also removed because they had greater than 60% of missing data. The final PEAR-2 dataset consisted of 856 metabolites (non-imputed data) which were included in the rest of the QC steps and in the analysis. Imputed dataset (imputation was done using the K-nearest neighbors (KNN) algorithm) was only used to conduct one of the QC steps (principal component analysis (PCA)).

PCA:

The first ten principal components (PCs) explain about 70% of the variability in the PEAR-2 metabolomics data **(Table S1)**. Based on the first three PCs which explain a total of 35.8% of the variability**,** there was no separation among the PEAR-2 observations (n=379) included in this study. However, four outliers have been identified **(Figure S2)**.

Standard Euclidean distance (SED):

SED is used to identify participants that are outliers based on their metabolic states using the pairwise SED between those participants. SED between each pair of participants (pairwise SEDs) and the SED between each participant and the estimated mean were calculated. Based on the SED values, 11 participants had outlying metabolic states (having the largest SED values) compared to the rest. Further investigation of those participants demonstrated that 5 of them had greater than 10% missing metabolomics data (the average missing was 8.6%). Also, 3 of those 5 participants had outlying/ extreme (greater than 3 standard deviations (SDs)) lipid (triglycerides, LDL and HDL) values as shown in **Table S2**.

Bland-Altman (BA):

BA assesses the concordance of the metabolomics data between pairs of participants, particularly those within specified groups [6]. A linear regression fit is generated between the values to identify/ flag any outlying values. To conduct this QC step, the PEAR-2 dataset was categorized into eight different groups: male participants aged < 50 years old with body mass index (BMI) < 30 kg/m^2 (n=39), male participants aged < 50 years old with BMI ≥ 30 kg/m^2 (n=44), male participants aged ≥ 50 years old with BMI < 30 kg/m^2 (n=74), male participants aged ≥ 50 years old with BMI ≥ 30 kg/m^2 (n=56), female participants aged < 50 years old with BMI < 30 kg/m^2 (n=50), female participants aged < 50 years old with BMI ≥ 30 kg/m^2 (n=49), female participants aged ≥ 50 years old with BMI < 30 kg/m^2 (n=26) and female participants aged ≥ 50 years old with BMI ≥ 30 kg/m^2 (n=41). Participant was flagged if greater than 20% of the metabolites' values for this participant were also flagged as outliers (participants within the same group are expected to have similar metabolites' values). Metabolite was flagged if greater than 5% of the participants' values for this metabolite were also flagged as outliers. The measures used to determine the outlying values were Pearson residuals, DFFITS and Cooks D. Based on these measures and on the BA plots, no participants were flagged, whereas 23 metabolites were flagged **(Table S3)**.

Coefficient of variation (CV):

CV assesses the consistency of the metabolomics data across participants and is calculated by dividing the SD by the mean for each metabolite. The higher the metabolite's CV value, the higher is the variability of that metabolite across participants. The top 10% of the metabolites with the largest CV values (exceeding the CV cutoff of 0.823, n=37) were flagged **(Table S4)**.

Figure S1. Consort diagram showing the participants included in this study.

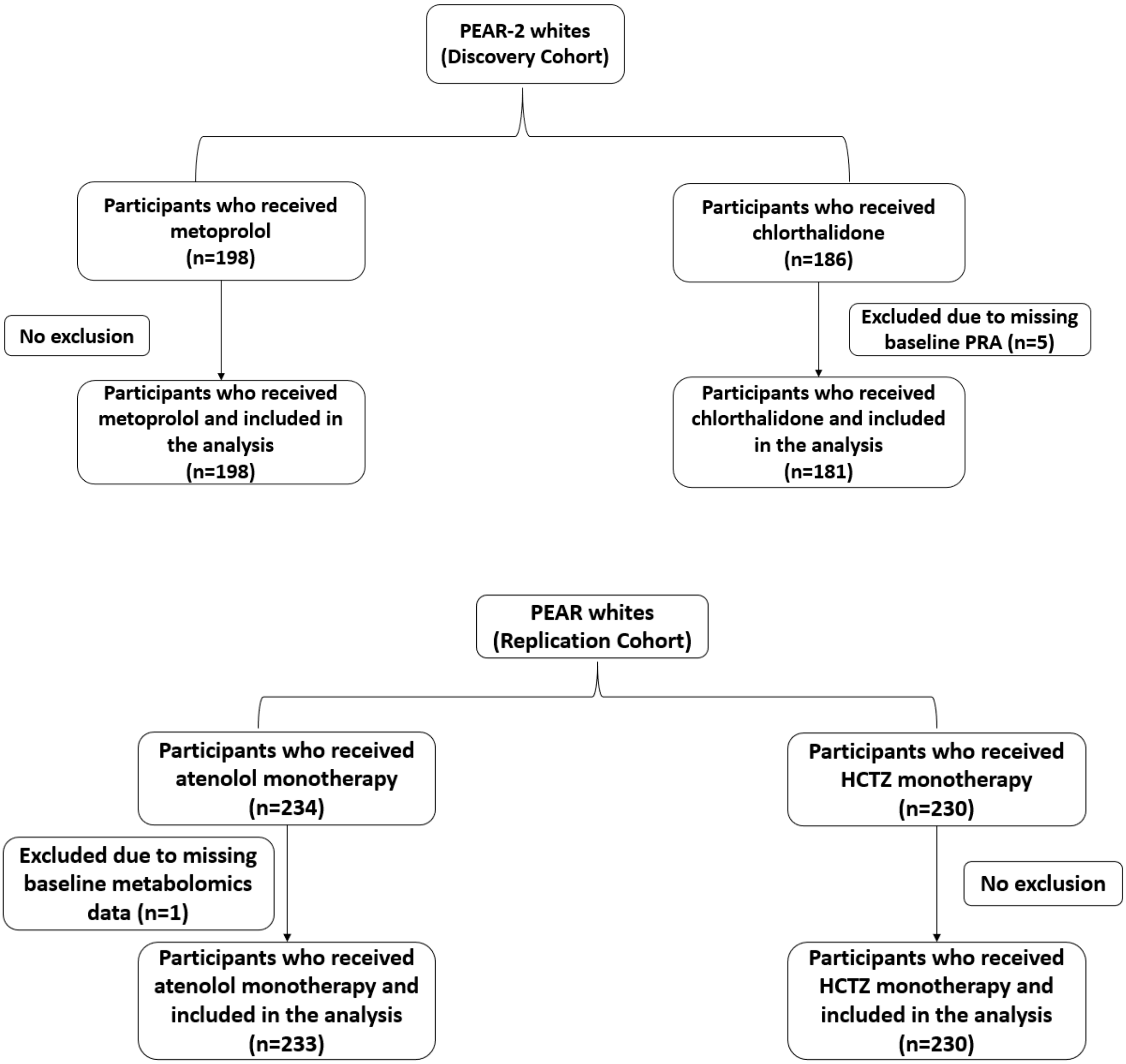

Figure S1. Consort diagram showing the participants included in this study. Abbreviations: PEAR, Pharmacogenomic Evaluation of Antihypertensive Responses; HCTZ, hydrochlorothiazide.

Figure S2. PCA scatter plots showing clustering of the PEAR-2 samples (n=379) based on the first three PCs.

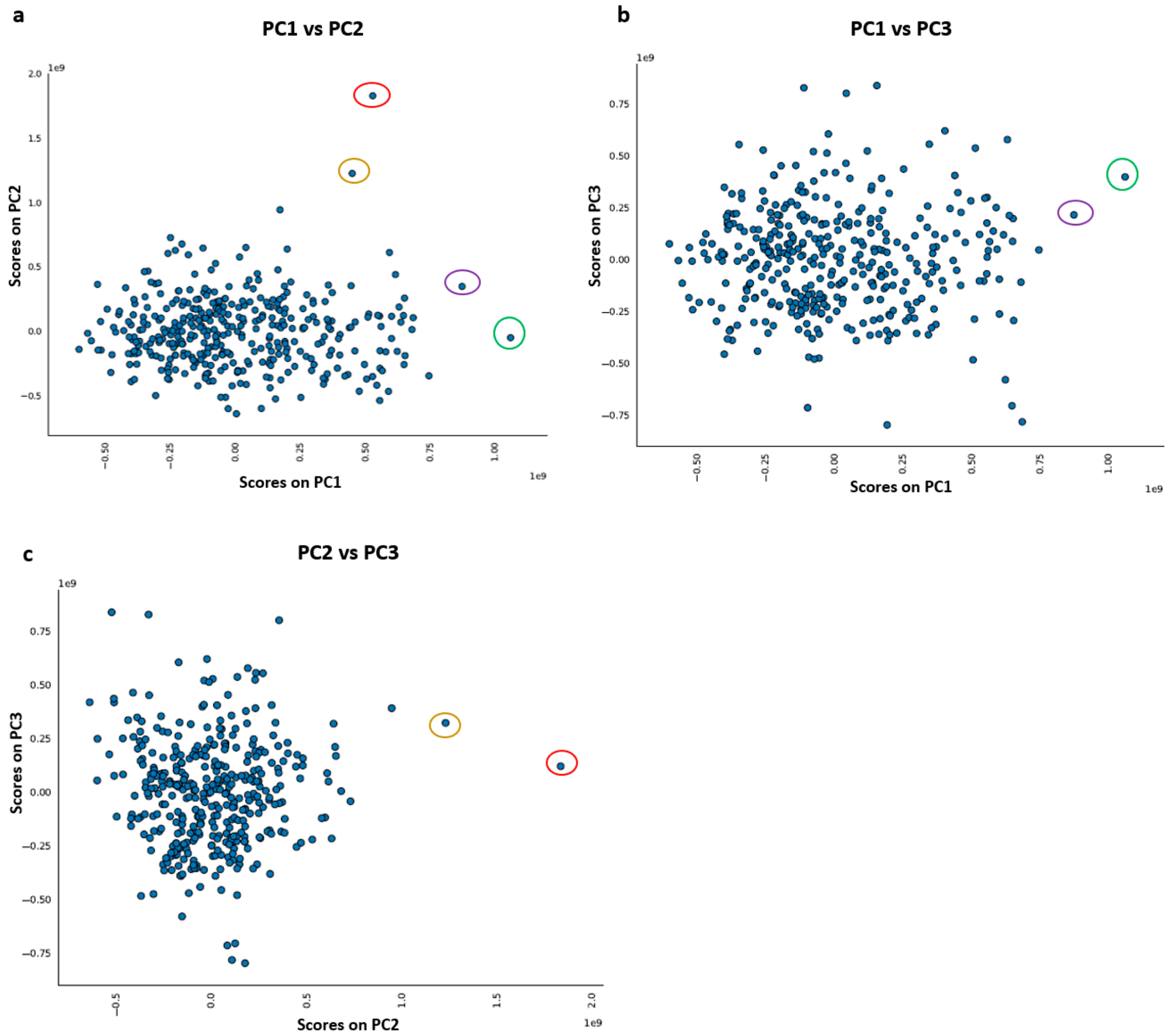

Figure S2. PCA scatter plots showing clustering of the PEAR-2 samples (n=379) based on a) PC1 vs. PC2, b) PC1 vs. PC3 and c) PC2 vs. PC3. PC1, PC2 and PC3 explain 13.8%, 12.2% and 9.8% of the variability in the metabolomics data, respectively. The scatterplots show four outliers (in red, brown, purple and green circles). Abbreviations: PCA, principal component analysis; PEAR, Pharmacogenomic Evaluation of Antihypertensive Responses; PC, principal component.

Table S1. Percent of variability in metabolomics data explained by each one of the first 10 PCs.

PC	% of variability explained
PC1	13.8%
PC2	12.2%
PC3	9.8%
PC4	7.5%
PC5	5.4%
PC6	5.2%
PC7	4.3%
PC8	3.9%
PC9	3.2%
PC10	2.9%

Abbreviation: PC, principal component.

Table S2. The 11 PEAR-2 participants with the largest SEDs.

Samples	Mean SED	% Missing Metabolites	Triglycerides Z-scores	LDL Z-scores	HDL Z-scores
**Participant 1**	54.8	12.9*	−0.7	−0.1	4**
**Participant 2**	48.7	6.4	−0.3	1.1	−1.2
**Participant 3**	41.2	7.6	−0.5	−0.02	1.8
**Participant 4**	35.1	4.6	−0.2	1.6	−0.5
**Participant 5**	33.7	3.2	−0.4	−1.1	−0.4
**Participant 6**	33.1	11.2*	3.4**	−3.8**	−2.2
**Participant 7**	32	11.4*	9.4**	−2.6	−2.1
**Participant 8**	31.7	9.3	0.6	−0.5	0.3
**Participant 9**	30.3	18.7*	−0.7	−0.4	2.5
**Participant 10**	29.7	10.4*	0.3	1.2	0.3
**Participant 11**	28.9	7.7	−0.5	0.7	−0.7

*These samples had > 10% missing metabolomics data.

**Participants with these samples had outlying/ extreme (>3 SDs) lipid values.

Abbreviations: PEAR, Pharmacogenomic Evaluation of Antihypertensive Responses; SED, standard Euclidean distance; LDL, low density lipoprotein; HDL, high density lipoprotein; SD, standard deviation.

Table S3. The 23 metabolites flagged by BA plots and measures.

Metabolite	Classification*	Pathway
**Acetylcarnitine**	Lipid	Fatty Acid Metabolism (Acyl Carnitine)
**Alanine**	Amino Acid	Alanine and Aspartate Metabolism
**Arachidonate (20:4n6)**	Lipid	Polyunsaturated Fatty Acid (n3 and n6)
**Arginine**	Amino Acid	Urea cycle; Arginine and Proline Metabolism
**Betaine**	Amino Acid	Glycine, Serine and Threonine Metabolism
**Creatine**	Amino Acid	Creatine Metabolism
**Creatinine**	Amino Acid	Creatine Metabolism
**Glycerophosphorylcholine (GPC)**	Lipid	Phospholipid Metabolism
**Histidine**	Amino Acid	Histidine Metabolism
**1-Oleoyl-2-linoleoyl-GPI (18:1/18:2)***	Lipid	Phospholipid Metabolism
**Lactosyl-N-palmitoyl-sphingosine**	Lipid	Sphingolipid Metabolism
**Linoleoyl ethanolamide**	Lipid	Endocannabinoid
**Lysine**	Amino Acid	Lysine Metabolism
**1-Oleoyl-GPE (18:1)**	Lipid	Lysolipid
**Myristate (14:0)**	Lipid	Long Chain Fatty Acid
**1-Palmitoleoylglycerol (16:1)***	Lipid	Monoacylglycerol
**1-Palmitoyl-2-gamma-linolenoyl-GPC (16:0/18:3n6)***	Lipid	Phospholipid Metabolism
**Palmitate (16:0)**	Lipid	Long Chain Fatty Acid
**Phenylalanine**	Amino Acid	Phenylalanine and Tyrosine Metabolism
**Threonine**	Amino Acid	Glycine, Serine and Threonine Metabolism
**Trigonelline (N'-methylnicotinate)**	Cofactors and Vitamins	Nicotinate and Nicotinamide Metabolism
**Urea**	Amino Acid	Urea cycle; Arginine and Proline Metabolism
**1-Palmitoylglycerol (16:0)**	Lipid	Monoacylglycerol

*Metabolites were classified based on the human metabolome database superclass classification http://www.hmdb.ca/classyfication.Abbreviations: BA, Bland-Altman.

Table S4. The top 10% of the metabolites with the largest CV values (n=37).

Metabolite	Classification*	Pathway
**N-Carbamoylalanine**	Amino Acid	Alanine and Aspartate Metabolism
**Oleoyl ethanolamide**	Lipid	Endocannabinoid
**Phenol sulfate**	Amino Acid	Tyrosine Metabolism
**Pregnen-diol disulfate***	Lipid	Pregnenolone Steroids
**Pyridoxate**	Cofactors and Vitamins	Vitamin B6 Metabolism
**Sphingosine**	Lipid	Sphingosines
**Trigonelline (N'-methylnicotinate)**	Cofactors and Vitamins	Nicotinate and Nicotinamide Metabolism
**Tryptophan betaine**	Amino Acid	Tryptophan Metabolism
**cis-3,4-methyleneheptanoyl carnitine**	NA	NA
**sulfate of piperine metabolite C16H19NO3 (2)***	NA	NA
**X – 11470**	NA	NA
**X – 11478**	NA	NA
**4-allylcatechol sulfate**	NA	NA
**X – 12462**	NA	NA
**X – 12543**	NA	NA
**1-Stearoyl-GPC (18:0)**	NA	NA
**X – 15245**	NA	NA
**X – 21310**	NA	NA
**indoleacetoylcarnitine***	NA	NA
**X – 23680**	NA	NA
**3-Hydroxy-5-cholestenoic acid**	Lipid	Sterol
**3-Methylglutarylcarnitine (2)**	Amino Acid	Lysine Metabolism
**3-Phenylpropionate (hydrocinnamate)**	Amino Acid	Phenylalanine and Tyrosine Metabolism
**3b-Hydroxy-5-cholenoic acid**	Lipid	Secondary Bile Acid Metabolism
**4-Androsten-3beta,17beta-diol disulfate (1)**	Lipid	Steroid
**4-Androsten-3beta,17beta-diol monosulfate (1)**	Lipid	Steroid
**5-HETE**	Lipid	Eicosanoid
**5Alpha-androstan-3alpha,17alpha-diol monosulfate**	Lipid	Steroid
**1-Linoleoyl-2-linolenoyl-GPC (18:2/18:3)***	Lipid	Phospholipid Metabolism
**Decanoylcarnitine**	Lipid	Fatty Acid Metabolism (Acyl Carnitine)
**Glycochenodeoxycholate**	Lipid	Primary Bile Acid Metabolism
**Glycolithocholate sulfate***	Lipid	Secondary Bile Acid Metabolism
**Glycoursodeoxycholate**	Lipid	Secondary Bile Acid Metabolism
**Hexanoylcarnitine**	Lipid	Fatty Acid Metabolism (Acyl Carnitine)
**Inosine 5'-monophosphate (IMP)**	Nucleotide	Purine Metabolism, (Hypo)Xanthine/Inosine containing
**Mannitol/sorbitol**	Carbohydrate	Fructose, Mannose and Galactose Metabolism
**N-Acetyltaurine**	Amino Acid	Methionine, Cysteine, SAM and Taurine Metabolism

*Metabolites were classified based on the human metabolome database superclass classification http://www.hmdb.ca/classyfication.Abbreviations: CV, coefficient of variation; NA, not applicable.

Table S5. The 48 metabolites nominally associated with the baseline Log PRA in PEAR-2 European Americans with $P < 0.01$.

Metabolite	Classification	Pathway	HMDB	Estimate ± SE	P-value
**N-acetylcarnosine**	Peptide	Dipeptide Derivative	HMDB12881	0.27 ± 0.08	0.001
**Gamma-glutamylglutamine**	Peptide	Gamma-glutamyl Amino Acid	HMDB11738	-0.08 ± 0.03	0.007
**Malate**	Energy Metabolite	TCA Cycle	HMDB00156	0.24 ± 0.07	0.001
**Succinylcarnitine**	Energy Metabolite	TCA Cycle	NA	-0.16 ± 0.06	0.0064
**1-(1-enyl-palmitoyl)-2-linoleoyl-GPE (P-16:0/18:2)***	Lipid	Plasmalogen	NA	0.05 ± 0.02	0.001
**1-(1-enyl-palmitoyl)-2-oleoyl-GPC (P-16:0/18:1)***	Lipid	Plasmalogen	NA	0.07 ± 0.02	0.003
**Cortisol**	Lipid	Steroid	HMDB00063	0.12 ± 0.04	0.001
**Cortisone**	Lipid	Steroid	HMDB02802	0.14 ± 0.05	0.0075
**5Alpha-androstan-3alpha,17alpha-diol monosulfate**	Lipid	Steroid	NA	0.1 ± 0.04	0.0087
**Myristoylcarnitine**	Lipid	Fatty Acid Metabolism (Acyl Carnitine)	HMDB05066	0.15 ± 0.05	0.002
**Decanoylcarnitine**	Lipid	Fatty Acid Metabolism (Acyl Carnitine)	HMDB00651	0.08 ± 0.03	0.0044
**Suberoylcarnitine**	Lipid	Fatty Acid Metabolism (Acyl Carnitine)	NA	0.09 ± 0.03	0.007
**3-Hydroxybutyrylcarnitine (1)**	Lipid	Fatty Acid Metabolism (Acyl Carnitine)	HMDB13127	0.09 ± 0.03	0.0087
**Palmitoylcarnitine**	Lipid	Fatty Acid Metabolism (Acyl Carnitine)	HMDB00222	0.15 ± 0.06	0.0099
**1-Palmitoyl-2-oleoyl-GPI (16:0/18:1)***	Lipid	Phospholipid Metabolism	NA	0.13 ± 0.04	0.0028
**1-Palmitoyl-2-palmitoleoyl-GPC (16:0/16:1)***	Lipid	Phospholipid Metabolism	NA	0.15 ± 0.05	0.003
**1-Stearoyl-2-oleoyl-GPE (18:0/18:1)**	Lipid	Phospholipid Metabolism	NA	0.13 ± 0.05	0.0068
**1-palmitoleoyl-GPC (16:1)***	Lipid	Lysolipid	HMDB10383	0.1 ± 0.03	0.002
**1-Palmitoyl-GPE (16:0)**	Lipid	Lysolipid	HMDB11503	0.17 ± 0.06	0.005
**1-Arachidonoyl-GPC (20:4n6)***	Lipid	Lysolipid	HMDB10395	-0.19 ± 0.07	0.004
**1-Oleoyl-GPE (18:1)**	Lipid	Lysolipid	HMDB11506	0.34 ± 0.12	0.0068
**10-Heptadecenoate (17:1n7)**	Lipid	Long Chain Fatty Acid	HMDB60038	-0.09 ± 0.03	0.003
**Glycerol**	Lipid	Glycerolipid Metabolism	HMDB00131	0.11 ± 0.04	0.0072
**1-Palmitoleoylglycerol (16:1)***	Lipid	Monoacylglycerol	NA	0.21 ± 0.08	0.0087
**Maleate**	Lipid	Fatty Acid, Dicarboxylate	HMDB00176	0.08 ± 0.03	0.009
**3-Hydroxydecanoate**	Lipid	Fatty Acid, Monohydroxy	HMDB02203	0.11 ± 0.04	0.0078
**Cysteinylglycine**	Amino Acid	Glutathione Metabolism	HMDB00078	0.09 ± 0.03	0.002
**Phenylacetylglutamate**	Amino Acid	Phenylalanine and Tyrosine Metabolism	HMDB59772	0.08 ± 0.03	0.003
**N-acetyltyrosine**	Amino Acid	Phenylalanine and Tyrosine Metabolism	HMDB00866	0.12 ± 0.04	0.0057
**Gentisate**	Amino Acid	Phenylalanine and Tyrosine Metabolism	HMDB00152	0.06 ± 0.02	0.0083
**Imidazole propionate**	Amino Acid	Histidine Metabolism	HMDB02271	0.15 ± 0.05	0.0054
**Lysine**	Amino Acid	Lysine Metabolism	HMDB00182	-0.33 ± 0.11	0.0058
**Leucine**	Amino Acid	Leucine, Isoleucine and Valine Metabolism	HMDB00687	0.1 ± 0.03	0.006
**N-acetylvaline**	Amino Acid	Leucine, Isoleucine and Valine Metabolism	HMDB11757	0.16 ± 0.06	0.0098
**4-Guanidinobutanoate**	Amino Acid	Guanidino and Acetamido Metabolism	HMDB03464	0.08 ± 0.03	0.0075
**Pantothenate**	Cofactors and Vitamins	Pantothenate and CoA Metabolism	HMDB00210	-0.06 ± 0.02	0.0068
**Linoleoyl-arachidonoyl-glycerol (18:2/20:4) [2]**	unknown	unknown	NA	0.13 ± 0.04	0.002
**2,3-Dihydroxy-5-methylthio-4-pentenoate (DMTPA)**	unknown	unknown	NA	-0.07 ± 0.02	0.002
**4-Hydroxyphenylacetylglutamine**	unknown	unknown	NA	0.09 ± 0.04	0.0099
**X – 15503**	unknown	unknown	NA	0.11 ± 0.03	0.002
**X – 12851**	unknown	unknown	NA	0.12 ± 0.04	0.002
**X – 14056**	unknown	unknown	NA	0.06 ± 0.02	0.0025
**X – 24309**	unknown	unknown	NA	0.03 ± 0.01	0.004
**X – 21815**	unknown	unknown	NA	0.08 ± 0.03	0.004
**X – 17654**	unknown	unknown	NA	0.12 ± 0.04	0.0047
**X – 21607**	unknown	unknown	NA	-0.08 ± 0.03	0.0056
**X – 17354**	unknown	unknown	NA	0.09 ± 0.03	0.0073

**X – 23780**	unknown	unknown	NA	0.07 ± 0.03	0.0083

P-values were produced using linear regression analysis of each metabolite with the baseline log-transformed plasma renin activity (PRA) in PEAR 2 European Americans, with adjustment of age, sex and baseline systolic blood pressure (SBP). Abbreviations: PRA, plasma renin activity; PEAR, Pharmacogenomic Evaluation of Antihypertensive Responses; SE, standard error; NA, not applicable; TCA, tricarboxylic acid cycle; HMDB, Human Metabolome Database.

Table S6. Availability of the metabolites having significant or nominally significant associations with the baseline PRA from discovery phase (n=63) in PEAR.

Significant or Nominally Significant Metabolites in PEAR-2	Availability in PEAR
**Sphinganine-1-phosphate**	Present
**Sphingomyelin (d18:1/20:1, d18:2/20:0)**	Present
**Sphingosine-1-phosphate**	Present
**1-(1-enyl-palmitoyl)-2-linoleoyl-GPE (P-16:0/18:2)***	Present
**1-Palmitoyl-2-oleoyl-GPI (16:0/18:1)***	Present
**1-(1-enyl-palmitoyl)-2-oleoyl-GPC (P-16:0/18:1)***	Present
**1-Palmitoyl-2-palmitoleoyl-GPC (16:0/16:1)***	Present
**1-Palmitoleoyl-GPC (16:1)***	Present
**1-Arachidonoyl-GPC (20:4n6)***	Present
**1-Palmitoyl-GPE (16:0)**	Present
**1-Oleoyl-GPE (18:1)**	Present
**1-Stearoyl-2-oleoyl-GPE (18:0/18:1)**	Present
**Cortisol**	Present
**Cortisone**	Present
**5Alpha-androstan-3alpha,17alpha-diol monosulfate**	Present
**3-Hydroxybutyrylcarnitine (1)**	Present
**10-Heptadecenoate (17:1n7)**	Present
**Glycerol**	Present
**1-Palmitoleoylglycerol (16:1)***	Present
**Maleate**	Present
**Caprate (10:0)**	Present
**3-Hydroxydecanoate**	Present
**Cysteinylglycine**	Present
**N-acetyltyrosine**	Present
**Gentisate**	Present
**Imidazole propionate**	Present
**Lysine**	Present
**Leucine**	Present
**N-acetylvaline**	Present
**N-acetylglutamate**	Present
**Beta-hydroxyisovalerate**	Present
**Threonine**	Present
**4-Guanidinobutanoate**	Present
**Pantothenate**	Present
**Fumarate**	Present
**Malate**	Present
**N-acetylcarnosine**	Present
**Gamma-glutamylglutamine**	Present
**2,3-Dihydroxy-5-methylthio-4-pentenoate (DMTPA)**	Present
**X – 15503**	Present
**X – 12851**	Present
**X – 14056**	Present
**X – 24309**	Present
**X – 21815**	Present
**X – 17654**	Present

**X – 21607**	Present
**X – 23780**	Present
**Sphinganine**	Absent
**Palmitoylcarnitine**	Absent
**Myristoylcarnitine**	Absent
**Decanoylcarnitine**	Absent
**Succinylcarnitine**	Absent
**Suberoylcarnitine**	Absent
**Phenylacetylglutamate**	Absent
**4-Hydroxyphenylacetylglutamine**	Absent
**3-Hydroxybutyroylglycine**	Absent
**3-Hydroxystachydrine**	Absent
**1-Methyl-5-imidazoleacetate**	Absent
**Glucuronide of C10H18O2 (7)**	Absent
**Linoleoyl-arachidonoyl-glycerol (18:2/20:4) [2]**	Absent
**X – 12726**	Absent
**X – 12818**	Absent
**X – 17354**	Absent

Abbreviations: PRA, plasma renin activity; PEAR, Pharmacogenomic Evaluation of Antihypertensive Responses.

Table S7. The metabolites clustered with caprate, sphingosine-1-phosphate and 1-palmitoleoyl-GPC (16:1)*.

	Clustered Metabolite Name	Classification	Pathway	Degree
Caprate	**1-(1-enyl-palmitoyl)-2-oleoyl-GPC (P-16:0/18:1)***	Lipid	Plasmalogen	0.431155443
	**1-(1-enyl-palmitoyl)-2-linoleoyl-GPE (P-16:0/18:2)***	Lipid	Plasmalogen	0.43073798
	**1-(1-enyl-palmitoyl)-2-palmitoleoyl-GPC (P-16:0/16:1)***	Lipid	Plasmalogen	0.413949423
	**1-linoleoylglycerol (18:2)**	Lipid	Monoacylglycerol	0.428059948
	**1-linoleoyl-GPA (18:2)***	Lipid	Lysolipid	0.420636403
	**3-hydroxyhexanoate**	Lipid	Fatty Acid, Monohydroxy	0.228050454
	**p-cresol sulfate**	Amino Acid	Phenylalanine and Tyrosine Metabolism	0.417169248
	**Methylsuccinate**	Amino Acid	Leucine, Isoleucine and Valine Metabolism	0.224595138
	**methylsuccinoylcarnitine (1)**	Amino Acid	Leucine, Isoleucine and Valine Metabolism	0.186115211
	**alpha-CEHC sulfate**	Cofactors and Vitamins	Tocopherol Metabolism	0.156961604
	**Glucuronate**	Carbohydrate	Aminosugar Metabolism	0.198347442
	**X – 12824**	unknown	Unknown	0.248259443
	**X – 22475**	unknown	Unknown	0.216592745
	**X – 24551**	unknown	Unknown	0.179023517
	**X – 12729**	unknown	Unknown	0.166716922
	**X – 22764**	unknown	Unknown	0.147428036
	**X – 13431**	unknown	Unknown	0.096858956
Sphingosine-1-phosphate	**Clustered Metabolite Name**	**Classification**	**Pathway**	**Degree**
	**Sphinganine**	Lipid	Sphingolipid Metabolism	0.368056837
	**Sphingosine**	Lipid	Sphingolipid Metabolism	0.354593032
	**sphinganine-1-phosphate**	Lipid	Sphingolipid Metabolism	0.135935101
	**1-palmitoyl-2-stearoyl-GPC (16:0/18:0)**	Lipid	Phospholipid Metabolism	0.302372322
	**1-stearoyl-2-linoleoyl-GPI (18:0/18:2)**	Lipid	Phospholipid Metabolism	0.221130745

	**Phosphoethanolamine**	Lipid	Phospholipid Metabolism	0.282640297
	**choline phosphate**	Lipid	Phospholipid Metabolism	0.203683279
	**Choline**	Lipid	Phospholipid Metabolism	0.131382677
	**5alpha-androstan-3beta,17alpha-diol disulfate**	Lipid	Steroid	0.222502942
	**5alpha-androstan-3alpha,17beta-diol monosulfate (2)**	Lipid	Steroid	0.099659612
	**leukotriene B4**	Lipid	Eicosanoid	0.178309248
	**Malonate**	Lipid	Fatty Acid Synthesis	0.064994991
	**adenosine 5'-monophosphate (AMP)**	Nucleotide	Purine Metabolism, Adenine containing	0.344258294
	**Adenosine**	Nucleotide	Purine Metabolism, Adenine containing	0.205261485
	**N6-succinyladenosine**	Nucleotide	Purine Metabolism, Adenine containing	0.199480006
	**Guanosine**	Nucleotide	Purine Metabolism, Guanine containing	0.287798715
	**inosine 5'-monophosphate (IMP)**	Nucleotide	Purine Metabolism, (Hypo)Xanthine/Inosine containing	0.259437531
	**Orotate**	Nucleotide	Pyrimidine Metabolism, Orotate containing	0.228840762
	**Orotidine**	Nucleotide	Pyrimidine Metabolism, Orotate containing	0.212602335
	**S-methylcysteine**	Amino Acid	Methionine, Cysteine, SAM and Taurine Metabolism	0.297586187
	**Hypotaurine**	Amino Acid	Methionine, Cysteine, SAM and Taurine Metabolism	0.205558503
	**Aspartate**	Amino Acid	Alanine and Aspartate Metabolism	0.251274305
	**Anthranilate**	Amino Acid	Tryptophan Metabolism	0.117047705
	**4-hydroxyglutamate**	Amino Acid	Glutamate Metabolism	0.114535281
	**Maltose**	Carbohydrate	Glycogen Metabolism	0.261139141
	**Xylose**	Carbohydrate	Pentose Metabolism	0.146729377
	**X – 15486**	unknown	Unknown	0.330125603
	**X – 14658**	unknown	Unknown	0.286466056
	**X – 12815**	unknown	Unknown	0.18484338
	**X – 14626**	unknown	Unknown	0.167098785
	**X – 16124**	unknown	Unknown	0.159882032
	**X – 24540**	unknown	Unknown	0.159033657
	**X – 01911**	unknown	Unknown	0.141168019
**1-Palmitoleoyl-GPC (16:1)***	**Clustered Metabolite Name**	**Classification**	**Pathway**	**Degree**
	**1-stearoyl-2-arachidonoyl-GPC (18:0/20:4)**	Lipid	Phospholipid Metabolism	0.435507488
	**1-palmitoyl-2-linoleoyl-GPC (16:0/18:2)**	Lipid	Phospholipid Metabolism	0.426020776
	**1-palmitoyl-2-oleoyl-GPI (16:0/18:1)***	Lipid	Phospholipid Metabolism	0.410196262
	**1-palmitoyl-2-linoleoyl-GPE (16:0/18:2)**	Lipid	Phospholipid Metabolism	0.354602072
	**1-palmitoyl-2-oleoyl-GPC (16:0/18:1)**	Lipid	Phospholipid Metabolism	0.284882098
	**1-stearoyl-2-arachidonoyl-GPE (18:0/20:4)**	Lipid	Phospholipid Metabolism	0.259855236
	**1-palmitoyl-GPA (16:0)**	Lipid	Lysolipid	0.491730565
	**1-oleoyl-GPI (18:1)***	Lipid	Lysolipid	0.477106725
	**1-palmitoyl-GPG (16:0)***	Lipid	Lysolipid	0.476366627
	**1-arachidonoyl-GPE (20:4n6)***	Lipid	Lysolipid	0.374548776
	**1-oleoyl-GPE (18:1)**	Lipid	Lysolipid	0.34871557

**1-arachidonoyl-GPI (20:4)***	Lipid	Lysolipid	0.218519576
**2-palmitoylglycerol (16:0)**	Lipid	Monoacylglycerol	0.369694925
**1-palmitoylglycerol (16:0)**	Lipid	Monoacylglycerol	0.244802209
**1-oleoyl-3-linoleoyl-glycerol (18:1/18:2)**	Lipid	Diacylglycerol	0.440218274
**10-undecenoate (11:1n1)**	Lipid	Medium Chain Fatty Acid	0.280975373
**N-acetyltyrosine**	Amino Acid	Phenylalanine and Tyrosine Metabolism	0.269064849
**dopamine sulfate (2)**	Amino Acid	Phenylalanine and Tyrosine Metabolism	0.177584345
**1-methylhistidine**	Amino Acid	Histidine Metabolism	0.427924467
**2-hydroxybutyrate/2-hydroxyisobutyrate**	Amino Acid	Methionine, Cysteine, SAM and Taurine Metabolism	0.220083479
**betaine**	Amino Acid	Glycine, Serine and Threonine Metabolism	0.159774895
**ribitol**	Carbohydrate	Pentose Metabolism	0.250111883
**N-acetylglucosamine/N-acetylgalactosamine**	Carbohydrate	Aminosugar Metabolism	0.206158734
**galactonate**	Carbohydrate	Fructose, Mannose and Galactose Metabolism	0.168680637
**X - 23293**	unknown	Unknown	0.238174521
**X - 23662**	unknown	Unknown	0.174205718
**X - 23765**	unknown	Unknown	0.168330145

Degree is the average of the values of correlations for the given metabolite in the cluster to other metabolites within that cluster. Abbreviations: TCA, tricarboxylic acid cycle.

Table S8. List of top metabolic pathways enriched in the pathway analysis.

Pathway	Total Number of Metabolites	Expected Hits	Observed Hits	P-value	FDR
**Sphingolipid metabolism**	21	0.511	5	9.88E-05	0.0083
**Purine metabolism**	65	1.58	5	0.0184	0.772
**Glycerophospholipid metabolism**	36	0.875	3	0.0544	1
**Pentose and glucuronate interconversions**	18	0.438	2	0.0689	1
**Phosphonate and phosphinate metabolism**					
**Ascorbate and aldarate metabolism**	8	0.194	1	0.179	1
**Taurine and hypotaurine metabolism**	8	0.194	1	0.179	1
**Glycine, serine and threonine metabolism**	33	0.802	2	0.19	1
**Arginine biosynthesis**	14	0.34	1	0.293	1
**Nicotinate and nicotinamide metabolism**	15	0.365	1	0.31	1
**Fatty acid biosynthesis**	47	1.14	2	0.318	1
**Histidine metabolism**	16	0.389	1	0.327	1
**Starch and sucrose metabolism**	18	0.438	1	0.36	1
**Pantothenate and CoA biosynthesis**	19	0.462	1	0.375	1
**beta-Alanine metabolism**	21	0.511	1	0.406	1
**Alanine, aspartate and glutamate metabolism**	28	0.681	1	0.501	1
**Inositol phosphate metabolism**	30	0.729	1	0.526	1
**Arachidonic acid metabolism**	36	0.875	1	0.592	1
**Pyrimidine metabolism**	39	0.948	1	0.622	1
**Tryptophan metabolism**	41	0.997	1	0.64	1
**Aminoacyl-tRNA biosynthesis**	48	1.17	1	0.699	1

Abbreviations: FDR, false discovery rate.

References:

1. Johnson, J.A.; Boerwinkle, E.; Zineh, I.; Chapman, A.B.; Bailey, K.; Cooper-DeHoff, R.M.; Gums, J.; Curry, R.W.; Gong, Y.; Beitelshees, A.L.; et al. Pharmacogenomics of antihypertensive drugs: rationale and design of the Pharmacogenomic Evaluation of Antihypertensive Responses (PEAR) study. *Am Heart J* **2009**, *157*, 442-449, doi:10.1016/j.ahj.2008.11.018.
2. Mehanna, M.; Gong, Y.; McDonough, C.W.; Beitelshees, A.L.; Gums, J.G.; Chapman, A.B.; Schwartz, G.L.; Johnson, J.A.; Turner, S.T.; Cooper-DeHoff, R.M. Blood pressure response to metoprolol and chlorthalidone in European and African Americans with hypertension. *J Clin Hypertens (Greenwich)* **2017**, *19*, 1301-1308, doi:10.1111/jch.13094.
3. Evans, A.M.; Br, B.; Liu, Q.; Mitchell, M.W.; Rj, R.; Dai, H.; Sj, S.; DeHaven, C.D.; Lad, M. High Resolution Mass Spectrometry Improves Data Quantity and Quality as Compared to Unit Mass Resolution Mass Spectrometry in High- Throughput Profiling Metabolomics. *Metabolomics* **2014**, *4*, 1-3.
4. Xia, J.; Sinelnikov, I.V.; Han, B.; Wishart, D.S. MetaboAnalyst 3.0--making metabolomics more meaningful. *Nucleic Acids Res* **2015**, *43*, W251-257, doi:10.1093/nar/gkv380.
5. Afgan, E.; Baker, D.; Batut, B.; van den Beek, M.; Bouvier, D.; Cech, M.; Chilton, J.; Clements, D.; Coraor, N.; Grüning, B.A.; et al. The Galaxy platform for accessible, reproducible and collaborative biomedical analyses: 2018 update. *Nucleic Acids Res* **2018**, *46*, W537-W544, doi:10.1093/nar/gky379.
6. Bland, J.M.; Altman, D.G. Statistical methods for assessing agreement between two methods of clinical measurement. *Lancet* **1986**, *1*, 307-310.
